# A commentary on “The association between diabetes mellitus and postoperative cognitive dysfunction: a systematic review and meta-analysis”

**DOI:** 10.1097/JS9.0000000000002933

**Published:** 2025-07-02

**Authors:** Qiaoying Liu, Hongying Xing, Xiaofang Du

**Affiliations:** a Department of General Surgery, Joint Logistics Force 969 hospital, Hohhot, Inner Mongolia, China; b Department of Nursing, Joint Logistics Support Force 969 hospital, Hohhot, Inner Mongolia, China; c Department of Gastroenterology, 969th Hospital of Joint Logistics Support Force, Hohhot, Inner Mongolia, Autonomous Region, China

*Dear Editor,*

We read with great interest the recent article titled “The association between diabetes mellitus and postoperative cognitive dysfunction: a systematic review and meta-analysis”^[1]^ published in the *International Journal of Surgery*. This study is to assess the relationship between the risk of postoperative cognitive dysfunction (POCD) and diabetes mellitus (DM). Compared to their non-diabetic counterparts, patients with diabetes have a markedly increased risk of POCD, according to the data. To elucidate the exact mechanisms underlying this link and investigate potential preventive measures for individuals with diabetes, more research is necessary. However, a number of significant issues need to be addressed in future research.

First, there is variation in the diagnostic criteria for POCD. The definitions and evaluation methods for POCD vary widely across different studies^[2]^. Some use screening tools like MMSE or MoCA, while others employ detailed neuropsychological tests^[3]^. This inconsistency can lead to misdiagnosis or missed diagnosis of POCD. Furthermore, some studies do not clearly specify the diagnostic threshold for POCD (such as Z-scores or SD changes), which further complicates the results.

Second, the follow-up time is inconsistent. The follow-up periods of the studies range from 1 day to 5 years, and the duration of POCD may vary depending on the follow-up period. Short-term follow-ups (such as within 1 week) may not be sufficient to differentiate between POCD and postoperative delirium (POD), while long-term follow-up studies are less common, potentially leading to an underestimation of the true incidence of POCD. This time difference makes it challenging to directly compare the results.

Third, there isn’t a thorough examination of anesthesia techniques. Although the study mentioned that anesthesia methods, such as general and local anesthesia, might affect the risk of POCD, it did not conduct a systematic analysis. Only a few studies provided detailed information on the type of anesthesia used, and the effects of different anesthetic drugs, such as propofol versus inhalation anesthesia, were not adjusted for. Key factors like anesthesia depth and intraoperative cerebral oxygen monitoring were also not discussed.

In summary, this study systematically analyzed the association between diabetes and POCD, but there are some design limitations, sample heterogeneity, and inconsistent diagnostic criteria. Future studies need to adopt a more unified definition of POCD, expand RCT evidence, and explore the mechanism and clinical intervention strategies in depth. All this was observed regarding the transparency in the reporting of Artificial Intelligence—the TITAN guideline^[4]^.

## Data Availability

No data was used in this Letter to the Editor.
